# Supplementary material for: Diet, Supplementation and Nutritional Habits of Climbers in High Mountain Conditions
Source: Nutrients. 2023 Sep 29;15(19):4219. doi: 10.3390/nu15194219 (PMC10574574; doi:10.3390/nu15194219)
Supplement: Supplementary file 1 [file nutrients-15-04219-s001.zip › nutrients-2625530-supplementary.pdf]

Questionnaire for assessing the dietary habits of climbers and Himalayan climbers in high altitude conditions

The questionnaire contains general questions about dietary habits in high mountain conditions (above 3,000 meters above sea level). I kindly ask you to mark your answers and write them in the dotted areas. I wish to thank you for sincere and honest completion of the questionnaire.

Basic data

1. Gender
   a) Female
   b) Male
2. Year of birth: ....
3. Body height [cm]: ....
4. Body weight [kg]: ....
5. How long have you been training in the sport you practice (alpine climbing)?
   c) 1-4 years
   d) 5-6 years
   e) 7-8 years
   f) 9-10 years
   g) More than 10 years
6. Affiliation with a sports club
   …………………………………………
7. Sports level (best performance - if applicable):
   Sport climbing: ....
   Bouldering: ....
   Mountaineering: ....
   Drytooling: ....
   Other:..
   ....

8. How often do you attend climbing training?
a) More than twice a week
b) Twice a week
c) Once a week
d) Less than once a week
e) I do not train climbing

9. How long are your climbing training sessions?
a) Less than 1h
b) 1 – 1.5 h
c) 1.5 – 2 h
d) 2 -3 h

10. How often do you attend supplementary training?
a) More than twice a week
b) Twice a week
c) Once a week
d) Less than once a week
e) I do not do supplementary training

11. How long do the follow-up trainings last?
a) Less than 1h
b) 1 – 1.5 h
c) 1.5 – 2 h
d) 2 -3 h

12. What sport do you practice as part of your supplementary training?
You may mark more than one answer.
e) Running
f) Cycling
g) Swimming
h) Gym
i) Other (what kind?).....................................

13. How often do you climb rocks outdoors during the rock-climbing season?
a) More than twice a week
b) Twice a week
c) Once a week
d) Less than once a week
e) I don't climb rocks outdoors

14. How often do you climb indoors?
a) More than twice a week
b) Twice a week
c) Once a week
d) Less than once a week
e) I do not climb indoors

15. How often do you train climbing in the mountains?
a) More than twice a week
b) Twice a week
c) Once a week
d) Less than once a week

16. How many times (on average) per year do you make expeditions to altitudes above 3,000 meters above sea level?

…………………………………………………

17. When was the last time you were above 3,000 meters above sea level?
(please provide the accurate date and duration if you were in the mountains during the last month).

…………………………………………………

18. Have you experienced acute altitude sickness so far?
    a) Yes
    b) No

    If so:
    Do you think that one of the causes of high mountain sickness may have been inadequate hydration?
    ………………………………
19. Do you observe any body weight loss after returning from trips?
    a) Yes
    b) No

Preparation for high altitude mountain climbing

1. Do you monitor your health by taking a blood count test before going on a high mountain expedition?
   a) Yes
   b) No
2. Do you do blood tests to determine your iron levels before going on an expedition?
   a) Yes
   b) No
3. Do you do blood tests to determine ferritin levels before you go on an expedition?
   a) Yes
   b) No
4. Do you consult with a nutritionist before going to the mountains?
   a) Yes
   b) No
5. Do you follow a diet other than your usual one (e.g., one that excludes a selected dietary ingredient, such as gluten-free, vegetarian)?
   a) Yes
   b) No
   If so, which one?
   ………………………………………

Feeding behavior during mountain action

Nutrition

1. How would you rate your appetite in the mountains above 3,000 meters above sea level?
   a) Very good
   b) Good
   c) Medium
   d) Small
   e) Lack of desire to eat meals
2. Does the way you eat in the mountains significantly differ from your usual diet?
   a) Yes
   b) No

3. What products do you most often take with you into the mountain action with a bivouac above base camp (multiple answers are possible)?
   a) Bars
   b) Energy gels
   c) Fruit mousses
   d) Jellybeans
   e) Chocolate
   f) Salty snacks
   g) Dried meat
   h) Dried legumes
   i) Canned meat
   j) Fish preserves
   k) Freeze-dried meals
   l) Other: (what?): ....

4. Do you reach for candy bars during mountain action?
   a) Yes
   b) No

5. What kind of candy bars do you choose most often?
   a) Cereal
   b) Dates
   c) High-protein
   d) Covered with chocolate
   e) Other (what?) ....
   f) I don't eat candy bars during mountain action

6. What foods do you most often consume after a mountain activity before camping in the mountains (multiple answers are possible)?
   a) Bars
   b) Energy gels
   c) Fruit mousses
   d) Jellybeans
   e) Chocolate
   f) Salty snacks
   g) Dried meat
   h) Dried legumes
   i) Canned meat
   j) Fish preserves
   k) Freeze-dried meals
   l) Other: (which?) ....

7. Is sweet taste (sweet carbohydrate snacks) your most preferred choice in the mountains? Is sweet taste (sweet carbohydrate snacks) your most preferred choice in the mountains?
    a) Yes
    b) No

    If not, which one?
    …………………………………………

8. Have you ever experienced fatigue with the sweet taste due to the high frequency and quantity of carbohydrate snacks consumed?
    a) Yes
    b) No

9. Which of the following food groups are in short supply during expeditions?
   It is possible to mark several answers.

a) Vegetables
b) Fruits
c) Whole grain products (whole grain bread, cereals, coarse groats, whole grain pasta, bran, etc.)
d) Purified grain products (white bread, white pasta, white rice, etc.)
e) Milk and dairy products
f) Meat
g) Meat preparations (canned meat, cold cuts, pate, etc.).
h) Fish
i) Eggs
j) Dry legumes
k) Butter and cream
l) Vegetable oils (olive oil, linseed oil, canola oil, etc.)
m) Seeds and nuts (walnuts, almonds, cashew, linseed, pumpkin seeds, sunflower seeds etc.)
n) Sugar and sweets

10. Which of the following food groups do you eat most often during your trips?
    It is possible to mark several answers.

a) Vegetables
b) Fruits
c) Whole grain products (whole grain bread, cereals, coarse groats, whole grain pasta, bran, etc.)
d) Purified grain products (white bread, white pasta, white rice, etc.)
e) Milk and dairy products
f) Meat
g) Meat preparations (canned meat, cold cuts, pate, etc.).
h) Fish
i) Eggs
j) Dry legumes
k) Butter and cream
l) Vegetable oils (olive oil, linseed oil, canola oil, etc.).
m) Seeds and nuts (walnuts, almonds, cashew, linseed, pumpkin seeds, sunflower seeds etc.)
n) Sugar and sweets

11. Kindly specify the FREQUENCY of consumption of the given product groups during expeditions by marking an X in the appropriate column.

	Several times a day	Once a day	Several times a week	Occasionally/ rarely	Never on an expedition
Vegetables					
Fruits					
Whole grain cereal products (whole grain bread, cereals, coarse groats, whole grain pasta, bran, etc.)					
Refined grain products (white bread, white pasta, white rice, etc.)					
Milk and milk products					
Meat					
Meat preparations (canned goods, cold cuts, pates, etc.)					

Fish					
Eggs					
Dry legumes					
Butter and cream					
Vegetable oils (olive oil, linseed oil, canola oil, etc.)					
Nuts and seeds (walnuts, almonds, cashews, linseed, pumpkin seeds, sunflower seeds, etc.)					
Sugar and sweets					

12. Are you willing to consume freeze-dried foods?
    a) Yes
    b) No

    If not, why?
    ……………………………………………

13. Which freeze-dried products do you consume the most?
    It is possible to mark several answers.
    a) Dinner dishes
    b) Soups
    c) Porridge / oatmeal / desserts
    d) Fruits
    e) Vegetables
    f) Cocktails
    g) Other

14. Have you ever experienced digestive discomfort after eating freeze-dried foods?
    a) Yes
    b) No

    If so, what did it involve?
    ……………………

15. Do you ever find yourself with not enough food in the mountains?
    a) Yes
    b) No

    If so, what is the reason?
    ……………………………………

16. Do you think the amount of food affects how you feel in the mountains?
    a) Yes
    b) No

17. Do you think the quality of food affects your well-being in the mountains?
    a) Yes
    b) No

18. How long does it take you to recover from a completed action in the mountains (when do you feel ready for the next action)?
    ……………………………………………

Hydration

19. What liquids do you take with you most often into the mountains?
    a) Water
    b) Isotonic beverages
    c) Juices
    d) Tea
    e) Coffee
    f) Others

    If others, please list them below:

    ………………………………………

20. How many liquids per day do you drink most often in the mountains?
    a) Less than 1 l
    b) 1-2 l
    c) 2-3 l
    d) 3-4 l
    e) More than 4 l

21. Do you think the amount of liquids you drink in the mountains is sufficient?
    a) Yes
    b) No

22. When melting water from a glacier, do you use water treatment tablets?
    a) Yes
    b) No

23. Do you use electrolytes as a water additive?
    a) Yes
    b) No

Supplements

24. Do you take protein nutrients with you to the mountains?
    a) Yes
    b) No
25. Do you take protein and carbohydrate nutrients with you to the mountains?
    a) Yes
    b) No
26. Do you take probiotics with you to the mountains?
    a) Yes
    b) No
27. Do you supplement with caffeine (e.g., in the form of an energy gel) in the mountains?
    a) Yes
    b) No
28. Do you supplement with beet juice in the mountains?
    a) Yes
    b) No
29. Do you supplement with omega-3 fatty acids in the mountains?
    a) Yes
    b) No
30. Do you use any other supplements in the mountains?
    a) Yes
    b) No

    If so, which ones?
    ……………………………………………
